# The patient statute (Law No. 15,378/2026) and the redefinition of medical authority: autonomy, dignity, and legal certainty at the bedside

**DOI:** 10.1590/1806-9282.20260777

**Published:** 2026-10-16

**Authors:** Fábio Roberto Cabar, Bruno Alves Gaia

**Affiliations:** 1 Universidade de São Paulo, School of Medicine – São Paulo (SP), Brazil.; 2 Universidade Paulista – Campus Alphaville, Instituto de Ciências da Saúde – São Paulo (SP), Brazil.

The 21st century has consolidated an irreversible paradigmatic shift in the physician–patient relationship: the definitive decline of traditional Hippocratic paternalism in favor of strengthening the principle of autonomy^1^. In the national context, this historical evolution reaches its institutional and normative peak with the enactment of Law No. 15,378 of April 6, 2026, which establishes the Patient Statute in Brazil^2^. This legislative milestone assumes an extremely relevant role in the Brazilian healthcare ecosystem, as it transposes purely ethical and deontological guidelines into the realm of cogent legal obligations^2^. The manifest objective of this legislation is to resolve complex bioethical dilemmas and provide much-needed legal certainty to daily clinical practice, with special emphasis on end-of-life scenarios, where the preservation of human dignity must imperatively prevail over the mere artificial biological maintenance of organs and functions^2^.

Autonomy, understood under the bioethical aegis as the inalienable right to self-government and self-determination regarding one's own health, stands as the cornerstone of person-centered medicine^1^. However, the transition of the physician's historical role, from an "absolute guardian of life" to a "facilitator of autonomy," imposes a profound and latent moral tension at the bedside^1^. The apparent conflict between the duty of beneficence (acting for the patient's clinical well-being) and respect for the sovereignty of individual will becomes the epicenter of complex debates within healthcare institutions^1^. Although the Brazilian Code of Medical Ethics had already signaled this horizontalization in recent decades, the positive law brought by the 2026 Statute alters the level of the discussion, transforming conduct recommendations into binding normative mandates^3^.

This dilemma manifests with dramatic contours and greater intensity when the strictly technical convictions of the care team collide with the sovereign decision of the patient to refuse a specific treatment^3^. From this scenario emerges the critical concept of moral distress: the psychological and existential exhaustion experienced by the healthcare professional when compelled by force of law to follow a course of action that directly contradicts their clinical and technical perception of what would be the "best interest" of the patient^3^. It is imperative to understand, however, that in contemporary bioethics, beneficence cannot be exercised in an impositive or authoritarian manner; it must obligatorily be filtered and reframed through the prism of the values, beliefs, and biography of the individual experiencing the illness process^4^.

Faced with such impasses, the 2026 Patient Statute offers fundamental practical and operational dimensions for the peaceful resolution of conflicts within the hospital environment^2^. First, the new legislation grants strictly binding force to Advance Healthcare Directives, ensuring unequivocally that the individual's so-called "living will" is fully respected by the medical team and administrators, even in situations of supervening cognitive incapacity^5^. This legal determination acts as a powerful reducer of ethical clashes and exhausting litigation between the clinical body and the patient's family members^5^.

Furthermore, the legal text provides robust and explicit legal protection for the practice of orthothanasia^6^. Consequently, physicians who decide to suspend or abstain from introducing futile, invasive, or extraordinary treatments, characterized as therapeutic obstinacy or dysthanasia, are legally safeguarded, redirecting the assistance focus toward comprehensive palliative care focused on relieving physical, psychological, and spiritual suffering^6^. Such normative advancements culminate in greater predictability and legal protection for professional practice, defining clear boundaries for medical liability in the face of a patient's informed refusal, and mitigating the deep-seated fear of judicial litigation that historically impelled professionals toward the practice of defensive and tortuous medicine^2^.

The contemporary challenge posed to the medical community requires professionals to make a definitive postural transition: to abandon the traditional purely prescriptive and directive stance and adopt a genuinely dialogical posture, validating and welcoming the prospective autonomy of the patient^5^. Ultimately, the Brazilian Patient Statute does not diminish, weaken, or subvert the scientific and moral authority of the physician; on the contrary, it recontextualizes it and elevates it to a new level of humanistic sophistication^2^. Recognizing the biological limits of therapeutics, accepting the finitude inherent to the human condition, and allowing death to occur in its natural time constitute the highest and most noble expressions of comprehensive medical care^6^. The true ethical commitment of 21st-century medicine lies in the firm conviction that when a cure is no longer a technically achievable horizon, dignity must remain the supreme value guiding both the law and the art of caring^6^.

## Data Availability

The datasets generated and/or analyzed during the current study are available from the corresponding author upon reasonable request.
